# Genome-wide analyses of the mung bean NAC gene family reveals orthologs, co-expression networking and expression profiling under abiotic and biotic stresses

**DOI:** 10.1186/s12870-022-03716-4

**Published:** 2022-07-15

**Authors:** Rezwan Tariq, Ammara Hussain, Arslan Tariq, Muhammad Hayder Bin Khalid, Imran Khan, Huseyin Basim, Pär K. Ingvarsson

**Affiliations:** 1 Department of Plant Protection, Akdeniz University, 07070 Antalya, Turkey; 2 Department of Biotechnology, University of Okara, Punjab, 56300 Pakistan; 3 Department of Biosciences, COMSATS University Islamabad, Islamabad, Pakistan; 4 College of agronomy, Sichuan Agricultural University, Ya’an, China; 5 National Research Center of intercropping, The Islamia University of Bahawalpur, Bahawalpur, Pakistan; 6 State Key Laboratory of Grassland Agro-Ecosystem, Livestock Industry Innovation, Ministry of Agriculture, College of Pastoral Agriculture Science and Technology, Lanzhou, 730020 China; 7 Linnean Centre for Plan Biology, Department of Plant Biology, Swedish University of Agricultural Sciences, SE75007 Uppsala, Sweden

**Keywords:** Mung bean, NAC, Transcription factor, Phylogeny, Co-expression network, Gene ontology, Biological process

## Abstract

**Background:**

Mung bean is a short-duration and essential food crop owing to its cash prominence in Asia. Mung bean seeds are rich in protein, fiber, antioxidants, and phytonutrients. The NAC transcription factors (TFs) family is a large plant-specific family, participating in tissue development regulation and abiotic and biotic stresses.

**Results:**

In this study, we perform genome-wide comparisons of *VrNAC* with their homologs from Arabidopsis. We identified 81 NAC transcription factors (TFs) in mung bean genome and named as per their chromosome location. A phylogenetic analysis revealed that *VrNACs* are broadly distributed in nine groups. Moreover, we identified 20 conserved motifs across the *VrNAC*s highlighting their roles in different biological process. Based on the gene structure of the putative *VrNAC* and segmental duplication events might be playing a vital role in the expansion of mung bean genome. A comparative phylogenetic analysis of mung bean NAC together with homologs from *Arabidopsis* allowed us to classify *NAC* genes into 13 groups, each containing several orthologs and paralogs. Gene ontology (GO) analysis categorized the *VrNACs* into biological process, cellular components and molecular functions, explaining the functions in different plant physiology processes. A gene co-expression network analysis identified 173 genes involved in the transcriptional network of putative *VrNAC* genes. We also investigated how miRNAs potentially target VrNACs and shape their interactions with proteins. VrNAC1.4 (Vradi01g03390.1) was targeted by the Vra-miR165 family, including 9 miRNAs. Vra-miR165 contributes to leaf development and drought tolerance. We also performed qRT-PCR on 22 randomly selected *VrNAC* genes to assess their expression patterns in the NM-98 genotype, widely known for being tolerant to drought and bacterial leaf spot disease.

**Conclusions:**

This genome-wide investigation of *VrNACs* provides a unique resource for further detailed investigations aimed at predicting orthologs functions and what role the play under abiotic and biotic stress, with the ultimate aim to improve mung bean production under diverse environmental conditions.

**Supplementary Information:**

The online version contains supplementary material available at 10.1186/s12870-022-03716-4.

## Background

Plants produce edible organic substances from simple inorganic molecules that can help feed the growing human and livestock populations. However, plants are subject to environmental extremes such as light, temperature, nutrients deficiency, and biological challenges, e.g., pests and pathogens [1] that can severely impact yield and quality of plant products. The evolutionary arm-race between plants and environmental challenges has enabled plants to adapt stressful conditions allowing them to persist under various environmental conditions. Crop plants are facing a great range of environmental stresses, many of which are likely to occur concurrently. In order to overcome such stresses, plants encode a wide range of stress-responsive genes, most of which are known from detailed work in model plants such as Arabidopsis and rice [2]. Transcription factors (TFs) are genes that act as pivotal regulators of plant responses to abiotic stresses such as cold, drought, and salt [3]. The TFs are interacting with cis-elements in the promoter region of different stress-related genes, and TFs have been found to be acting as molecular switches for the transcription of their target genes. Approximately, ~ 7% of a typical plant genome encrypts putative TFs and these habitually belong to large gene families [1].

One such large family of transcription factors is the NAC family of TFs. These genes are characterized by the presence of a NAC domain and the first example of such gene was first reported in petunia [4]. The NAC domain is comprised of an N-terminal DNA-binding domain, a nuclear localization signal (NLS), and a C-terminal transcriptional activation domain (AD). The N-terminal region consists of a160 amino acid long conserved DNA domain, which can be further subdivided into five subdomains (A–E) [5] which are arranged in the order A > C > D > B > E. The subdomains A and C have been revealed to be important for protein structure stabilization [5]. The highly variable C-terminal region interacts with other transcription factors and likely plays role in different developmental functions [6]. NAC TFs have numerous functions in plants, i.e., the formation of plant shoots apical meristems [7], nutrient transfer, control of the cell cycle in the senescence process [8], plant stress response [7], regulation of plant innate immunity [9], and hormone signalling [10]. For example, *OsNAC4* is a key positive regulator of programmed cell death in plants [11] and overexpression of *OsNAC6* resulted in increased tolerance to blast disease in rice [12]. A number of NAC proteins may positively regulate plant defence responses by activating pathogenesis-related (PR) genes, inducing genes involved in mediating the hypersensitive response (HR) and programmed cell death at the infection site in resistant plant species [13]. *HvNAC6* was found to be induced under resistance conditions in barley in response to *Pseudomonas syringae*, *Botrytis cinerea* and *Alternaria brassicola* infections [14, 15]. Likewise, understanding the complex mechanism of drought and salinity tolerance is imperative for future agriculture production and, interestingly, numerous NAC genes have been identified to be involved in plant response to drought and salinity stress. In transgenic rice, *OsNAC2* and *OsNAC6* and *OsNAC10* genes have been shown to enhance drought and salinity tolerance [16]. Several genes are induced by drought and salt stresses in tolerant cultivars across a range of species, including *TaNAC69* and *TaNAC6* in wheat [17, 18] and *CarNAC3* in chickpea [19]. NAC genes have been identified from 166 species, e.g., there are 105 NAC genes in Arabidopsis [20], 151 in rice [21], 142 in grapevine [22], 163 in Populus [23], 113 in Japanese apricot [24], 63 in coffee [25], and 152 in soybean genome [26]. However, NAC genes have thus far not been well studied in mung bean.

Mung bean (*Vigna radiata L.*), a well-known pulse crop, belongs to the subfamily *Papilionoideae* of Fabaceae. It originated in Southeast Asia, where its progenitors occur wildly. This edible legume crop is being grown on more than 6 million ha worldwide (about 8.5% of the global pulse area) and consumed in large quantities across Asia [27]. Mung bean is widely cultivated in Asia, in dry regions of southern Europe, and in the warmer parts of Canada and the United States owing to the fact that this is a low-input crop, relatively drought-tolerant, and which has a short growth cycle (< 80 days) [28]. Mung bean is a cheaper source of carbohydrates, high-quality protein, folate, and iron compared to most other legumes. Due to the nutritional value and health benefits of mung bean for people in developing countries, there has been a large interest in developing genetic and genomic tools to enhance mung bean breeding [29]. The availability of a draft genome sequence has significantly enhanced the progress of downstream analyses using the mung bean genome sequences [30]. The NAC gene family has a diverse role in plant development and physiology, and it is, therefore, crucial to further investigate this gene family in mung bean. Here, we analyzed the chromosome localization, conserved motifs, genetic structure, and phylogenetic relationships, based on sequence data from 81 *VrNAC* TFs. Our results will facilitate follow-up studies aimed at functionally characterizing the NAC genes in mung bean.

## Results

### Identification of NAC family members in mung bean

The amino acid sequences of putative *VrNAC* TFs, having the conserved DNA binding domain of NAC, were used as an input in BLAST searches for mung bean. Therefore, BLAST results identified 81 putative *VrNAC* genes; each gene was interpreted as *VrNAC*1.1 to *VrNAC*11.3 according to their respective positions on the chromosomes in the mung bean genome. The putative *VrNAC* genes encoded predicted proteins, ranging from 106 to 973 AA (amino acids) with isoelectric point (pI) from 4.39 to 10.74, and molecular weights, ranging from 9168.2 to 106,654 Da. However, comprehensive information of all identified VrNAC proteins, including their accession numbers, gene length, and chromosome location is shown in Table S1.

### Chromosomal distribution of VrNAC genes and synteny analysis

**Fig. 1:**
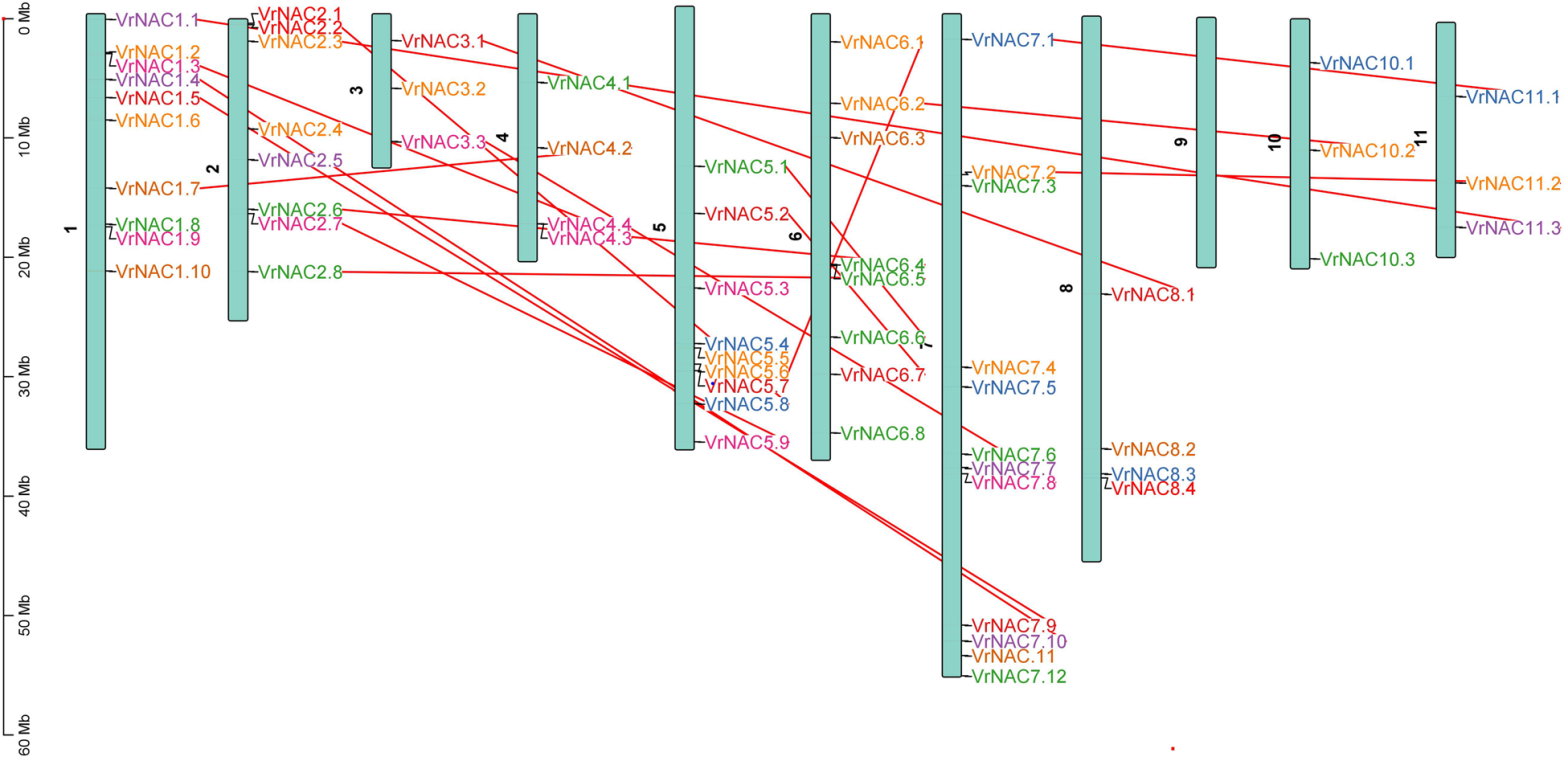
Distribution of *VrNAC* genes across the mung bean chromosomes. The length of each chromosome is given in Mbs; Lines linking different *VrNAC*s highlight segmental duplication events

To examine the *VrNACs* distribution on different chromosomes, the genomic sequences of *VrNACs* were used as a query to search against the mung bean genome to pinpoint the *VrNACs* distribution on chromosomes. The physical map positions revealed mapping of *VrNACs* on the mung bean chromosomes as per the location from short to long arm telomere (Fig. 1). Although, the majority of the chromosomes harbored *VrNAC* genes, the distribution was highly uneven, with a particularly high enrichment seen on chromosome 7 with 12 *VrNAC* genes; whereas chromosome 9 did not harbor any *VrNAC* genes at all. Other chromosomes with many *VrNAC* genes include chromosome 1 with 10 and chromosome 5 with 9 and chromosome 2 with 8 *VrNAC* genes. Chromosomes 3, 10, and 11 only harbored 3 *VrNAC* genes per chromosome.

**Fig. 2:**
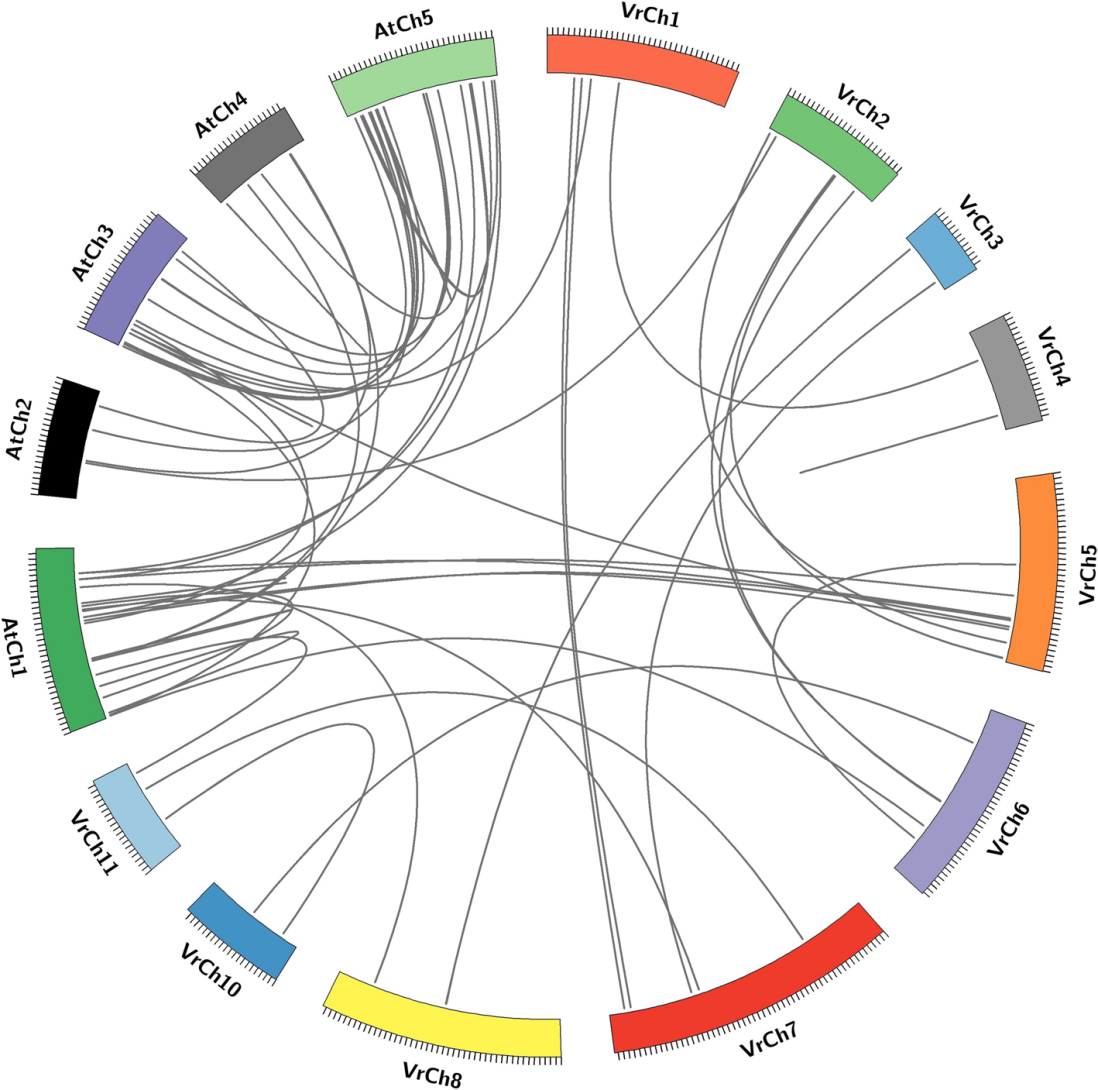
Synteny representation of the inter-chromosomal relationships of mung bean NACs and their evolutionary relationship with Arabidopsis. Black lines identify tandem and segmental duplications as well as orthologs pairs of NAC genes. The different color blocks represent different Arabidopsis and mung bean chromosomes

Duplication events play a vital role in plant evolution, such as tandem and segmental duplications are important processes resulting in genome expansion and increased complexity. We were able to infer duplication events in mung bean and Arabidopsis based on our phylogenetic analyses (Fig. 1; Fig. 2), providing important information regarding the function of VrNACs as orthologs, serve as the main source of functional predictions of genes through comparative analysis. Several segmental duplication events were identified in both the mung bean and Arabidopsis genomes, with tandem and segmental duplication events being more prominent in Arabidopsis, an observation that has already been described in earlier studies. However, no tandem duplication events were observed in this study. Duplication patterns were observed for a total of 15 segmental duplication pairs and suggest that segmental duplication events have played a vital role in the amplification of *VrNAC*s. Different orthologs combinations were identified between mung bean and Arabidopsis and overall 11 different orthologs were identified, likely to have similar functions in mung bean. The findings from our analyses of NAC gene orthologs in Arabidopsis and mung bean help illuminate how function may be assigned through the use of phylogenetic relationships between orthologous genes. However, the duplication events we inferred highlight segmental duplication as a driving force in the evolution of VrNACs and may thus be associated with future applications of the genes from the *VrNAC* family.

### Gene structural analysis

**Fig. 3:**
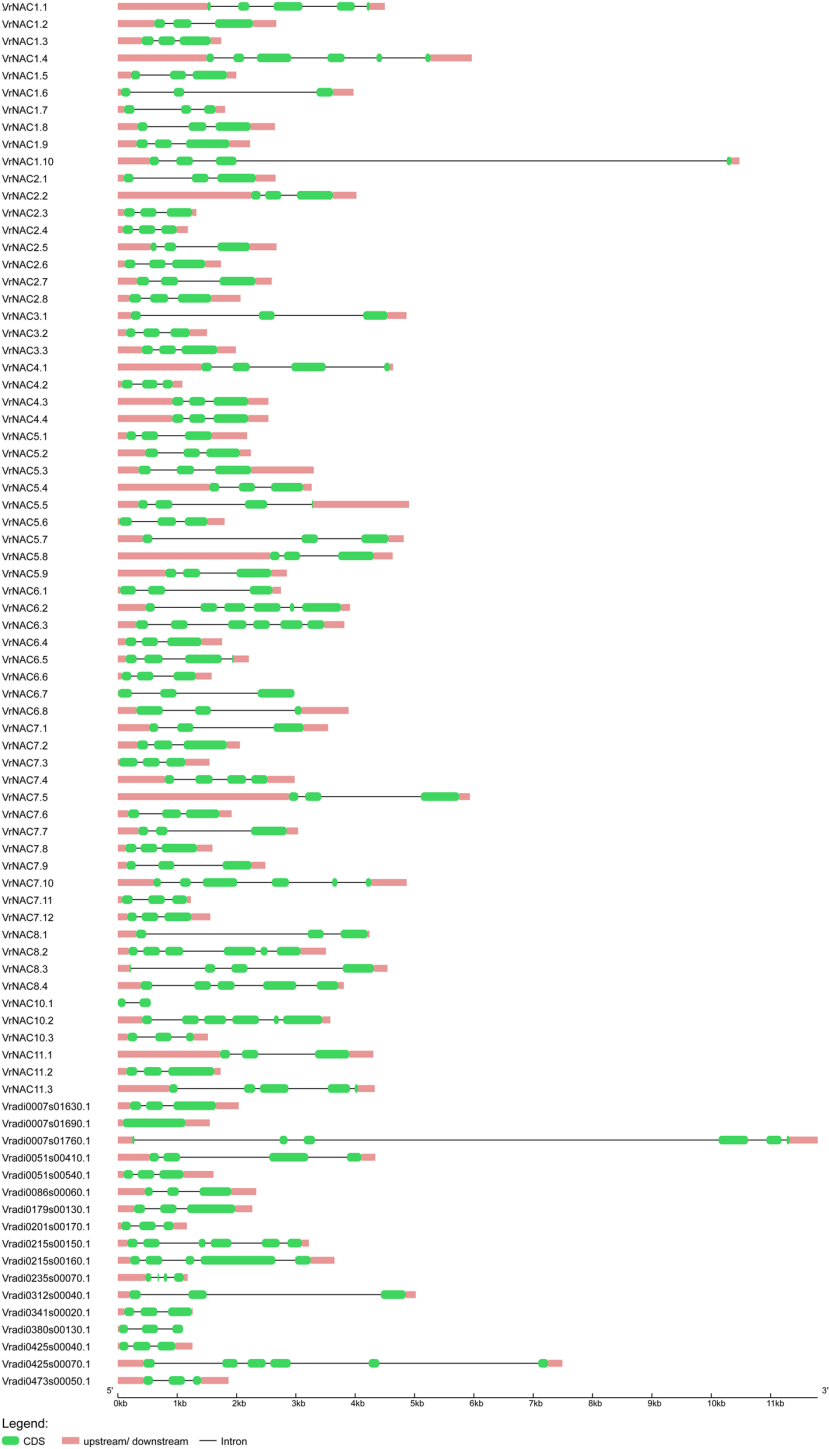
Gene structure analysis representing the exon/intron distributions in *VrNAC* TFs. The pink and green boxes represent UTRs and exons, respectively; while black lines indicate introns

During the evolution of multigene families, newly evolved copies are often evolving new gene functions, and this is also reflected in the diversification of gene structures in the *VrNAC* family. To further understand the structural diversity of different *VrNAC* genes, exon/intron sequences of all *VrNAC*s were used for to assess their structural organization using GSDS2.0 (http://gsds.gao-lab.org/) (Fig. 3). The results from the *VrNAC* gene structures show that the number of introns ranges from one to five, with one NAC gene (Vradi0007s01690.1) completely lacking introns.

### Phylogenetic classification and protein motif analysis of VrNACs

**Fig. 4:**
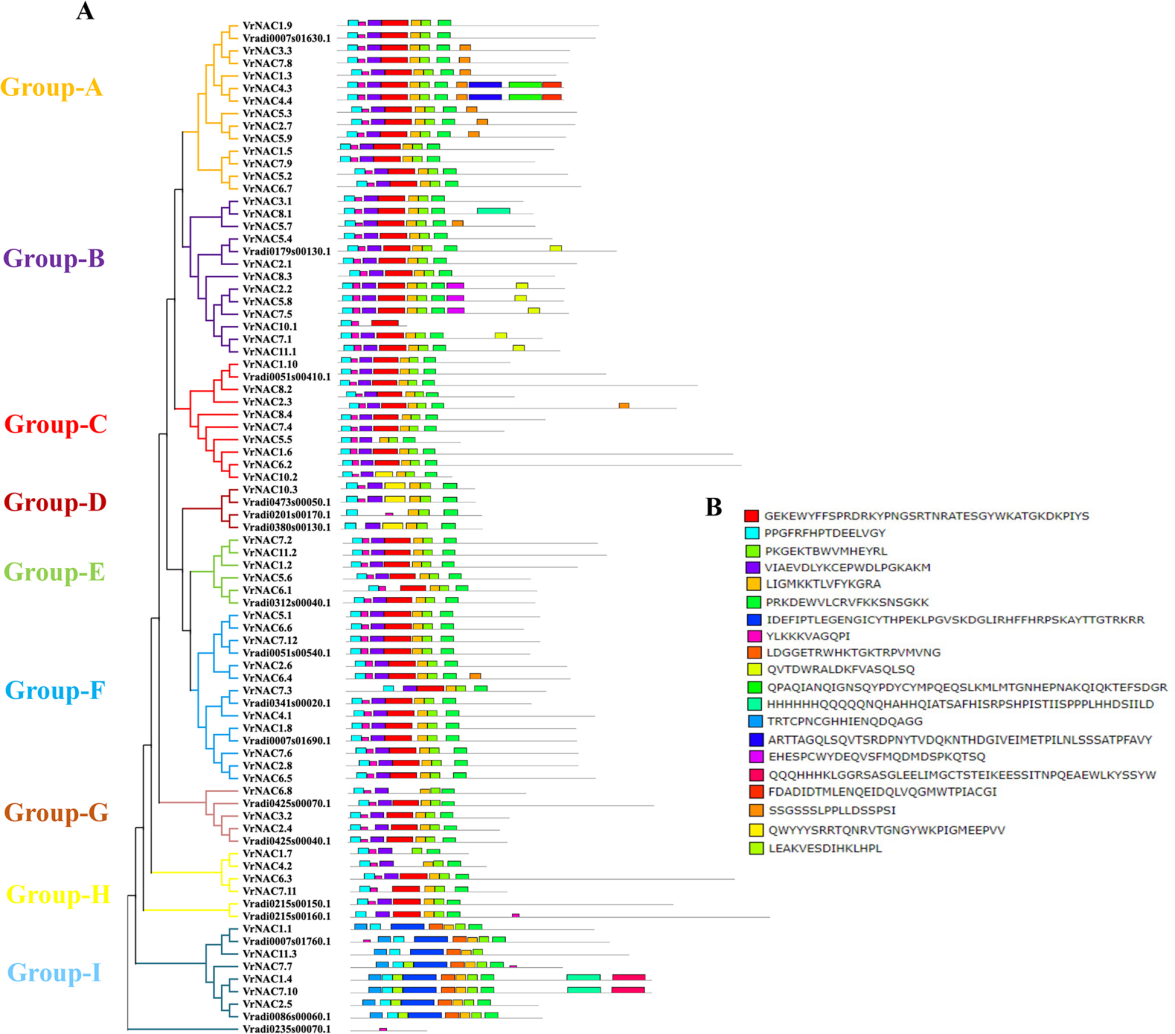
Phylogenetic analysis and conserved domain analysis of the *VrNAC* proteins from mung mean. **A** All *VrNAC* TFs are categorized into nine groups based on their protein sequences. **B** Conserved motifs of *VrNAC* TFs proteins as per the phylogenetic relationship. The conserved motifs were determined using MEME and each conserved motif is indicated by a different color; likewise, the length of each motif is displayed correspondingly

To explore the phylogenetic relationships among the 81 *VrNAC*s, we built a phylogenetic tree based on the *VrNAC* proteins using the Neighbour-Joining (NJ) method. This method was used as sequence lengths of the individual VrNACs, varied dramatically between different genes. The results indicate that the VrNAC family can be classified into 9 subfamilies, hereafter referred to as Group A to Group I (Fig. 4). Group A and Group B were the largest groups with 14 and 13 *VrNAC* proteins, respectively, followed by groups Group D and Group G with 4 and 5 proteins, respectively. These results suggest that *VrNAC* proteins may have played a critical role in the mung bean genome expansion.

We also analyzed putative conserved motifs in the *VrNAC*s using MEME (https://meme-suite.org/meme/) to further study the evolution of the *VrNAC* proteins. We identified 20 conserved motifs (1–20) from the *VrNAC* proteins. As expected, closely related genes, belonging to the same phylogenetic clade, mostly had similar motif compositions and only minor differences were observed within clades, suggesting that genes within clades are true paralogs. Based on the conservation of various domains, group A and group H contained a maximum of 11 conserved motifs, followed by group B, which contained 10 conserved motifs, group C contained 9 conserved motifs, group D, E, F, G, and H have the same 7 conserved motifs, i.e., 1, 2, 3, 4, 6, 18 and 20 motifs showed the high conservation of these motifs within clades. Remarkably, conserved motifs in the N-terminal of the *VrNAC* proteins are highly conserved for DNA-binding and similar motif compositions were shared within the clades. Such motifs conservation among the proteins suggests functional similarity and conserved motifs may thus indicate potential functional sites and that genes participate in inducing similar downstream functions.

### Comparative phylogenetic analysis of NAC between mung bean and Arabidopsis

**Fig. 5:**
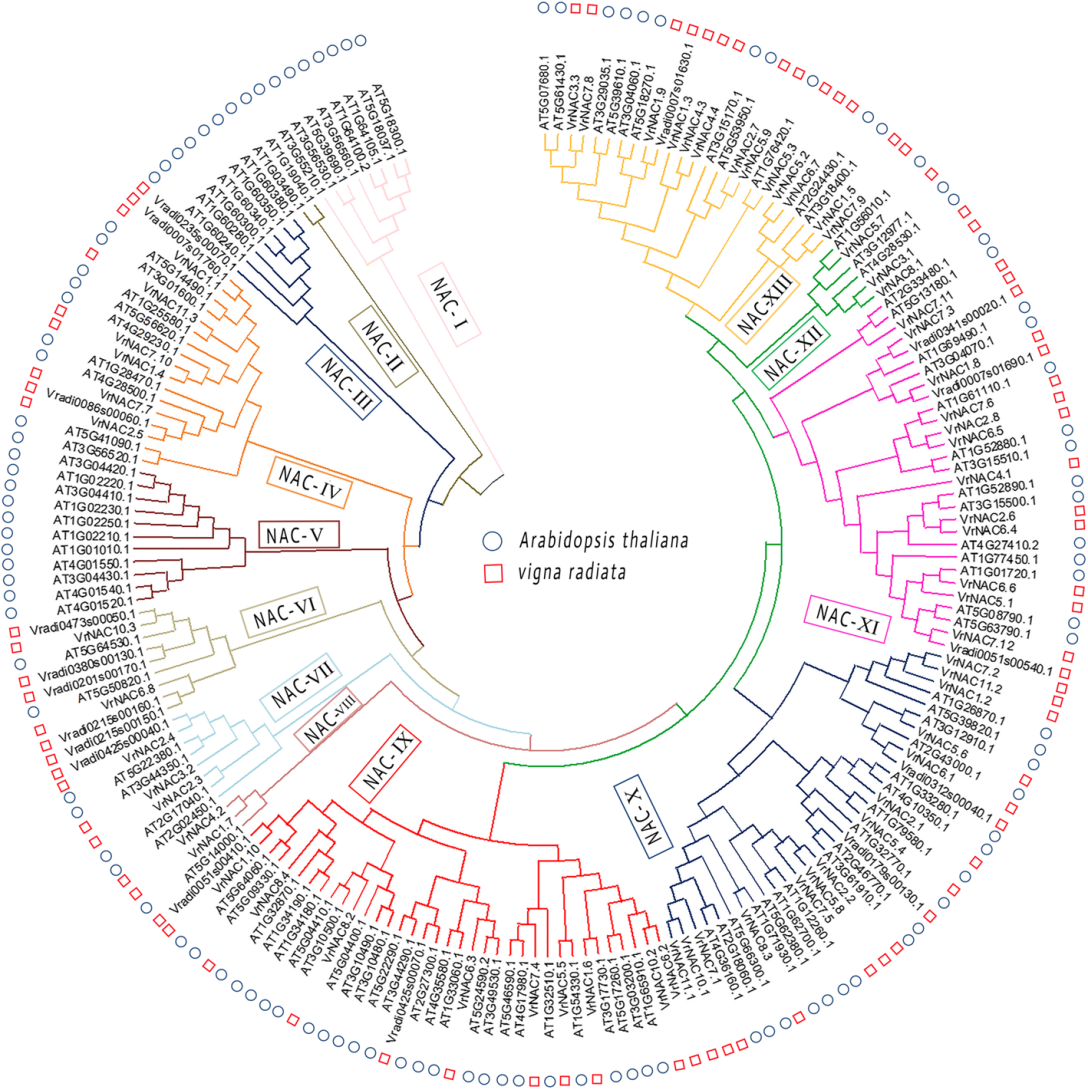
A comparative phylogenetic analysis based on the mung bean and Arabidopsis protein sequences of NACs. The Neighbor-joining (NJ) tree was constructed using Clustal Omega (https://www.ebi.ac.uk/Tools/msa/clustalo/) with the 1000 bootstrap replicates to assess tree reliability. NAC TFs from mung bean and Arabidopsis are classified into 13 different groups, highlighted by different colors in the tree

Exploring the comparative evolutionary relationship among different TFs between different plant species, a neighbor-joining phylogenetic tree was built from mung bean and Arabidopsis NAC proteins (Fig. 5). All members of NAC TFs from Arabidopsis and mung bean were classified into 13 subgroups, designated as NAC-I to NAC-XIII, respectively. NAC-IX constituted the prime clade with 36 *VrNAC* members followed by NAC-X which contains 33 *VrNAC* protein. The smallest clades, NAC-I, NAC-II and NAC-V, contained no genes from mung bean. The core objective of this comparative phylogenetic analysis was to detect putative orthologous genes between mung bean and Arabidopsis NACs. A key component of comparative genomics is to track the presence, structural characteristics, and functional similarities of orthologous genes across multiple genomes. Based on the inferred phylogenetic tree, *VrNAC* genes were comparatively assessed to their Arabidopsis orthologs. Different orthologous genes are present in the mung bean genome. For example, NAC-IX and NAC-XI have two orthologs, NAC-X have three orthologs while Group-XII and Group-XIII have one gene orthologous with Arabidopsis*.*

### Evolution collinearity of NAC genes between mung bean and other plant species

**Fig. 6:**
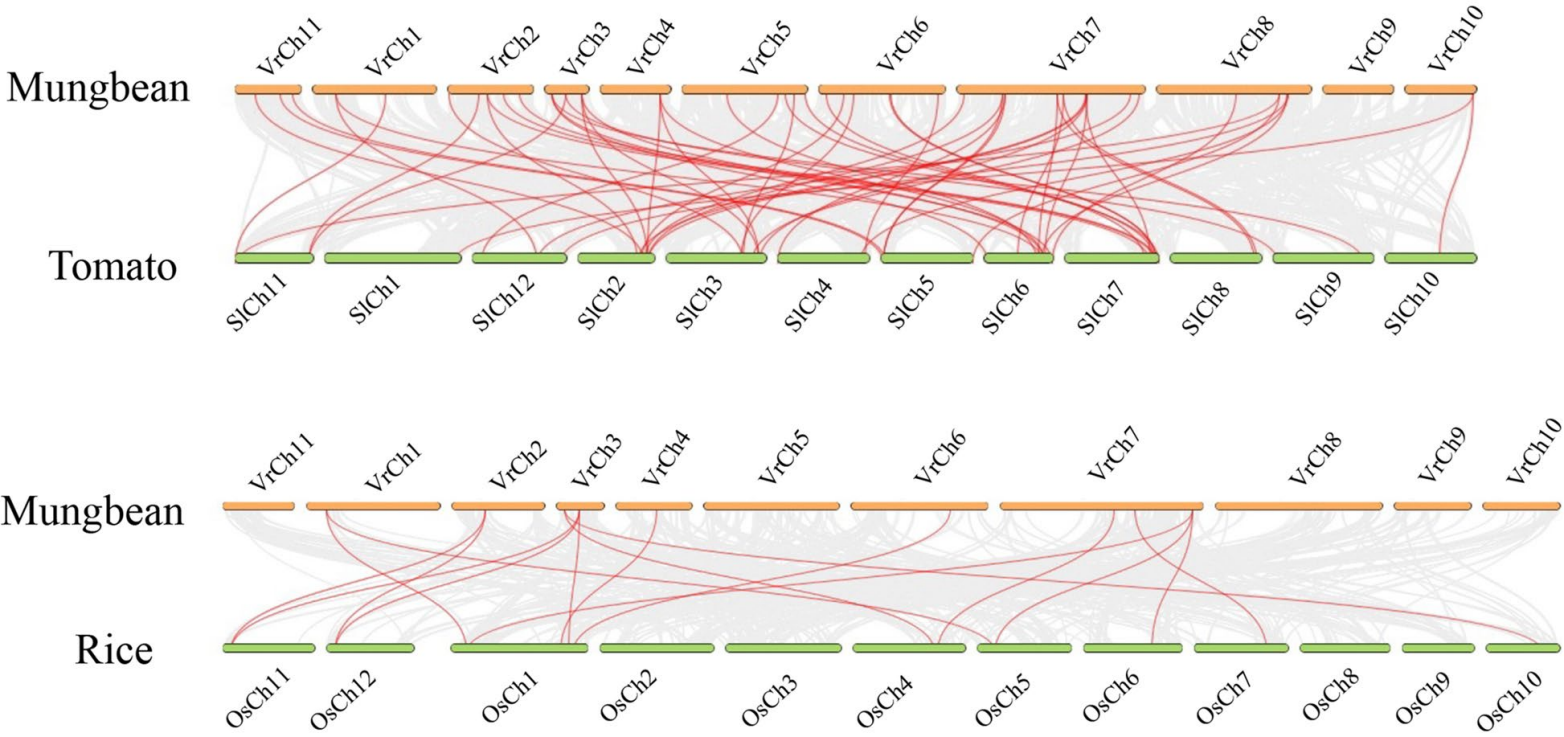
Collinearity analysis of *NAC* genes between mung bean and tomato or rice species. Gray lines indicate collinear blocks and red lines highlight homologous NAC gene pairs

We employed collinearity analysis to identify homologous genes and evolutionary relationships among genes. To explore the evolutionary relationship of *VrNAC*s with other plant species, we performed comprehensive collinearity analyses using rice and tomato (Fig. 6; Table S2). Tomato has many health-promoting compounds including vitamins, carotenoids and phenolic compounds, and exhibits 58 homologous pairs with mung bean. Rice is a monocot, belonging to the Poaceae family, often serves as a secondary model plant together with Arabidopsis and we identified 16 homologous pairs between rice and mung bean. The results indicate that NACs evolved from a common ancestor and have diversified across different plant species.

### Cis-element analysis of the VrNAC gene family

Cis-acting regulatory elements are the binding sites for a particular TF which determine the initiation or repression of transcription. As such, these cis-regulatory elements are the imperative gene structures in a genome. In the current study, we identified putative cis-regulatory elements to further inspect the probable functions of different NAC family genes in mung bean. This analysis was done using the PlantCARE database based on the 1 kb sequences immediately upstream of the *VrNACs* transcription start site.

**Fig. 7:**
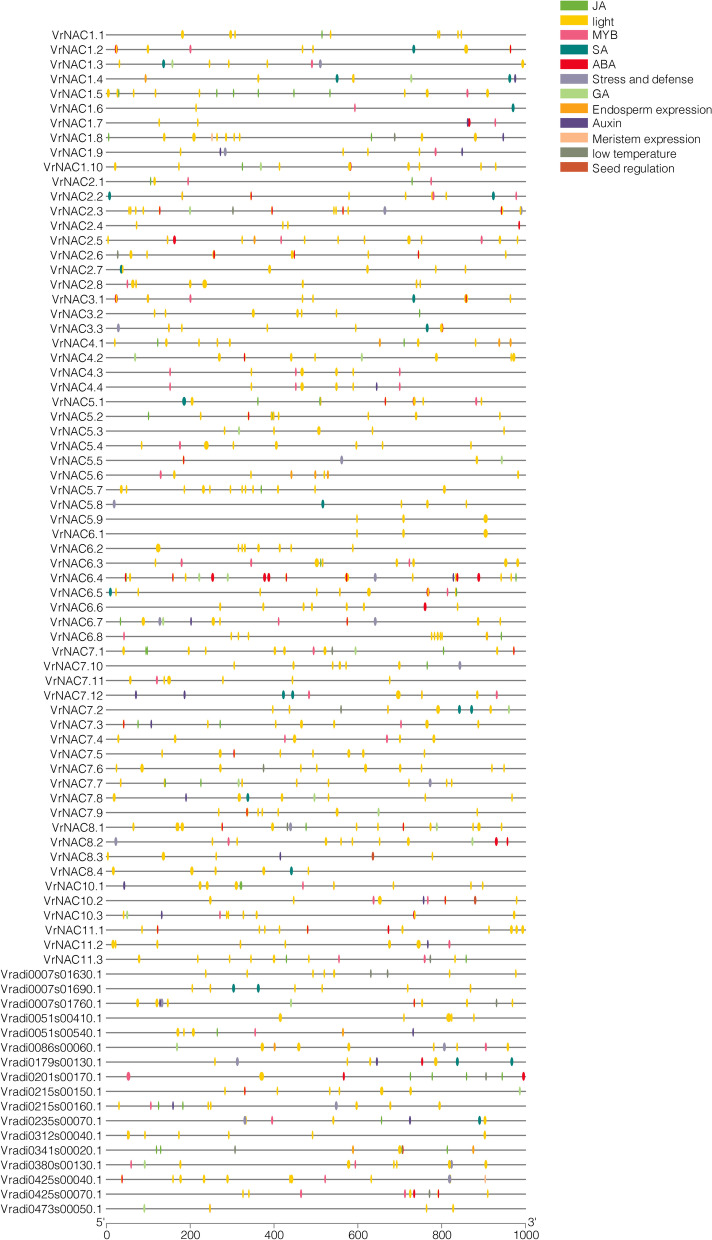
Cis-regulatory element analysis of *VrNAC* TFs. Cis-regulatory boxes involved in different environmental stress responses or as a response to different hormones are given in different colors

A total of 985 cis-regulatory elements, associated with different processes, i.e., abiotic and biotic stresses, developmental process, and light responsiveness, etc., were identified in the promoter regions of the *VrNAC* (Fig. 7; Table S3). Numerous cis-elements corresponding to tissue-specific expression, including root-specific, seed-specific, endosperm-specific, and meristem-specific expression were present in the *VrNAC* genes promoters. Similarly, several light-responsive cis-elements were revealed, broadly distributed in the *VrNAC*s promoter regions. Particularly, elements important for response to abiotic stress, including cold and dehydration-responsive elements, drought-responsive, low-temperature element, wound responsive, defense and stress-responsive elements were detected. Given the results, we can speculate that *VrNAC* TFs may counter different abiotic and biotic stresses and might have many prospective functions in enhancing the stress resistance in mung bean.

### Comprehensive miRNA targeting *VrNACs*

Micro-RNAs, miRNAs, are a global gene regulatory mechanism that help control gene expression under a large number of scenarios and miRNA-mediated gene regulation evolved more than 425 million years ago in the plant kingdom [31]. To further understand miRNA regulatory mechanisms, we identified putative miRNAs that target *VrNA*Cs (Table S4). The most highly targeted genes were VrNAC1.4 (Vradi01g03390.1), Vradi0007s01630.1, and Vradi07g17120.1 which contain 9, 6, and 5 miRNAs, while the least targeted genes, with one miRNA, are listed in the Table S4. Vra-miR164a, b, c, and d, were found to be the most abundant miRNAs collectively targeting 25 *VrNAC* genes. It’s noteworthy that Vra-miR164 has been shown to be involved in regulating drought and salt tolerance [32, 33].

### Gene ontology and co-expression regulation enrichment analysis

**Fig. 8:**
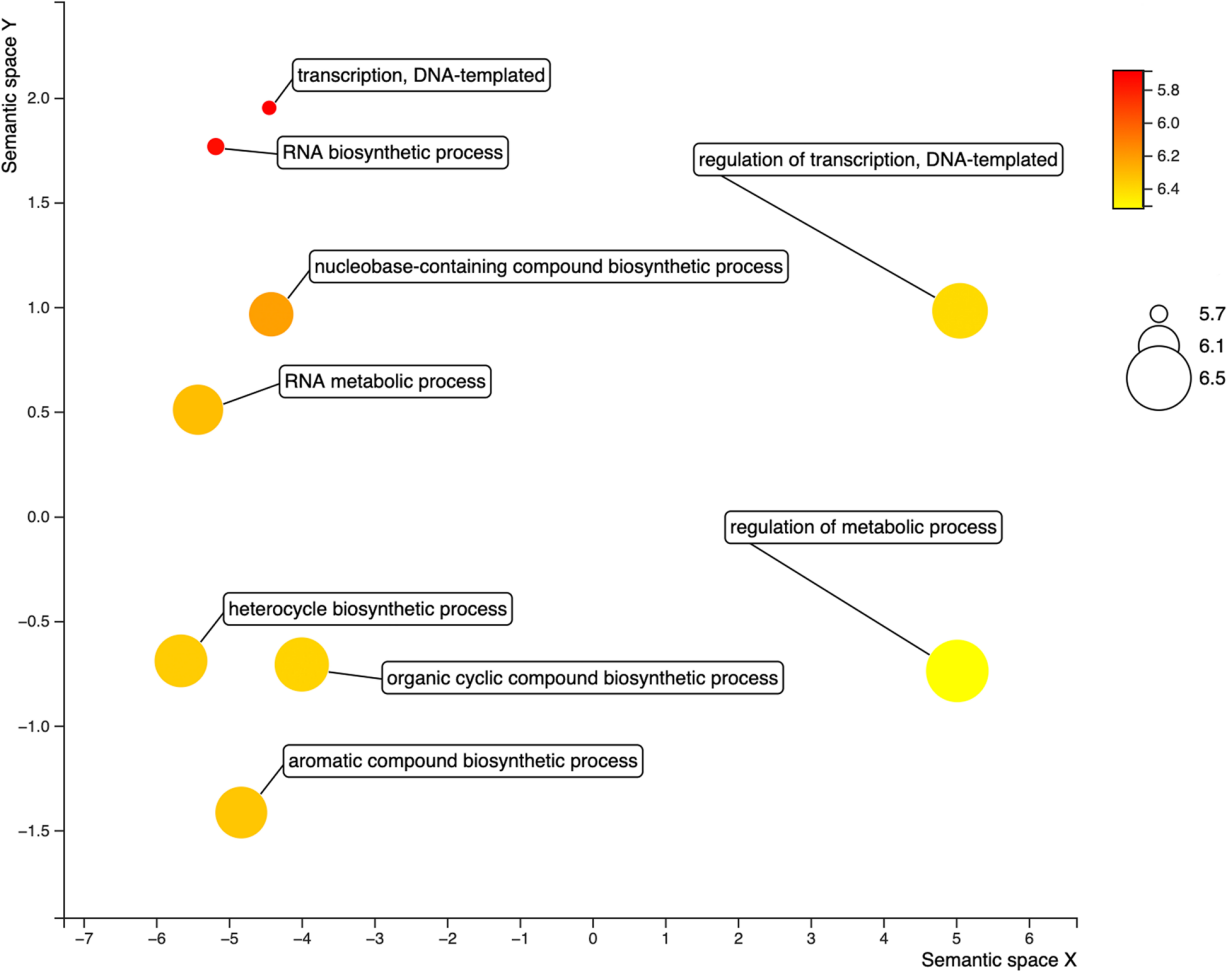
GO clustering exhibiting the involvement of *VrNAC* genes in different biological processes, participating in different mechanisms. The red to yellow color ribbon denote low to high enrichment patterns of *VrNAC* genes in different biological processes. The plot was produced using R (https://www.r-project.org)

Gene ontology (GO) analyses classified the 81 putative *VrNAC* TFs into three gene ontology categories, i.e., 1) biological process (47), 2) cellular component (6), and 3) molecular function (5), based on enrichments with *p* values < 0.05 (Table S5). A few important biological processes related to these GO terms are aromatic compound biosynthetic process (GO:0019438), organic cyclic compound biosynthetic process (GO:1901362), heterocycle biosynthetic process (GO:0018130), regulation of metabolic process (GO:0019222), and RNA metabolic process (GO:0016070) (Fig. 8). Given enrichment of *VrNAC*s in different GO terms, *VrNACs* can be inferred to play important roles across many different biological processes acting to maintain plant homeostasis in response to environmental influences.

**Fig. 9:**
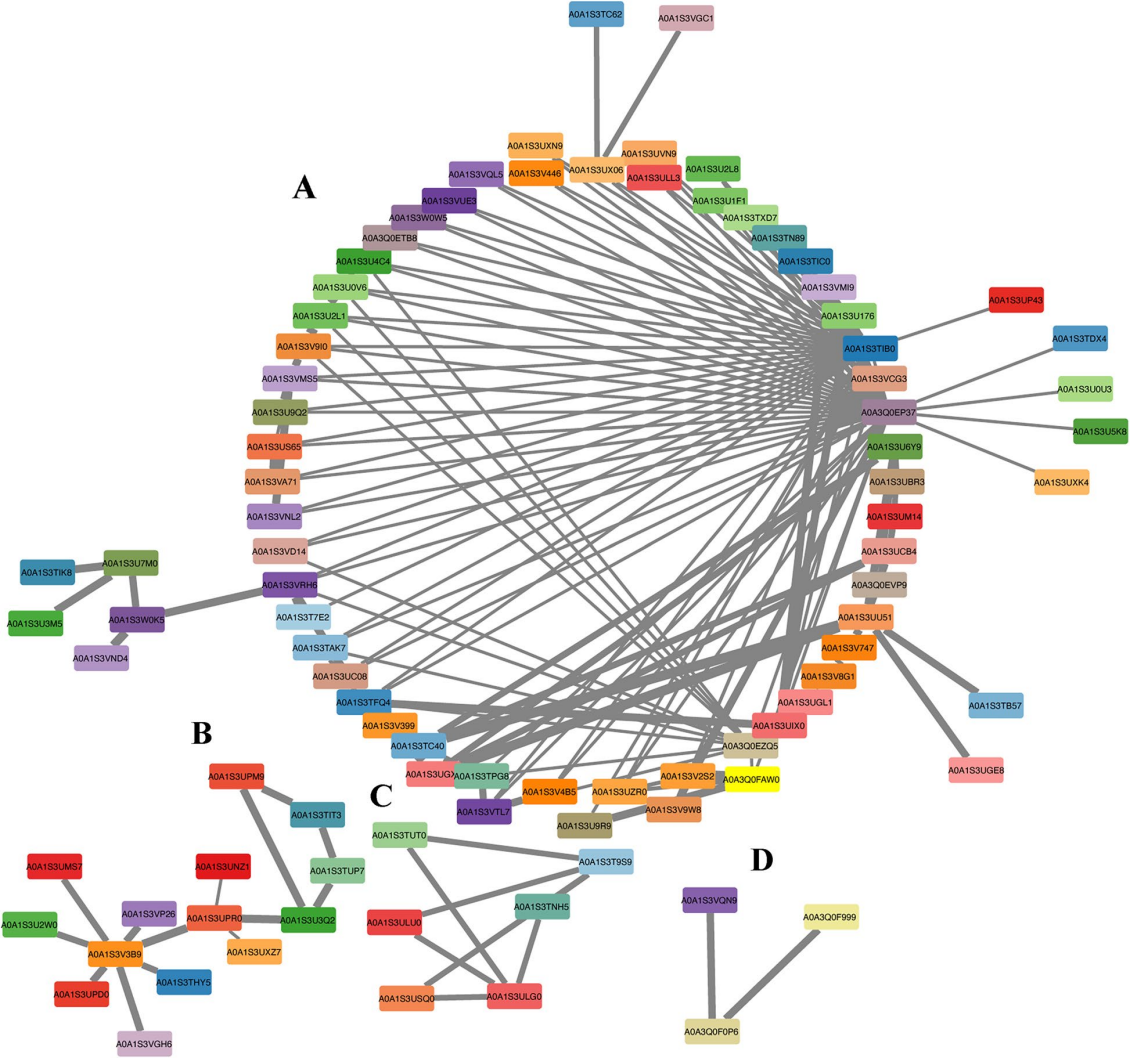
Co-expression network illustrating associations between different *VrNAC*s and other genes, involved in different abiotic and biotic stresses. The co-expression values of mung bean genes with annotations were retrieved from STRING database (https://string-db.org/) for making the correlation matrix and constructing the network among given genes via Cytoscape

Gene co-expression networks can be used to identify important regulatory genes and the regulatory roles of genes can be further investigated by the interaction between transcription factors (TFs) and their target genes. The complex network of genes and their positive and negative co-expression patterns thus control different biological and physiological mechanisms in the plant and is therefore important for plant responses to different environmental conditions. We constructed a co-expression network for *VrNAC*s based on a Pearson’s correlation coefficient (PCC) threshold of 0.4 [34], and we identified 173 genes in mung bean that are involved in the network together with the *VrNAC*s (Fig. 9; Table S6). We used Cytoscape (https://cytoscape.org/) to display the resulting networks of *VrNAC*s and their co-expressed genes. 37 *VrNAC* genes were included in the co-expression network with 100 other mung bean genes that may be participating in different regulatory mechanisms. For example, *VrNAC*2.6 is co-expressed with four genes, including three genes that belong to *VrNAC*s, i.e., *VrNAC*5.1(A0A1S3U6Y9), *VrNAC*6.6 (A0A1S3UCB4), and *VrNAC*7.12 (A0A1S3TPG8). Moreover, *VrNAC*2.2 (A0A1S3THY5) is co-expressed with 39 different genes, including A0A1S3U176 (Xylulose 5-phosphate/phosphate translocator, chloroplastic) and A0A1S3U2L1 (Ergosterol biosynthetic protein 28), A0A1S3U9Q2 (Vesicle transport protein), etc. Similarly, *VrNAC*8.4 is co-expressed with 13 genes, including A0A1S3VNL2 (Lipid phosphate phosphatase epsilon 2) and A0A3Q0EP37 (NAC domain-containing protein 40), for regulating the different physiological functions in mung bean. Our network analysis hence reveals genes that are regulating different stress response mechanisms via positive or negative co-expression patterns under different environmental conditions to maintain plant homeostasis.

### Expression pattern of VrNACs under stresses

**Fig. 10:**
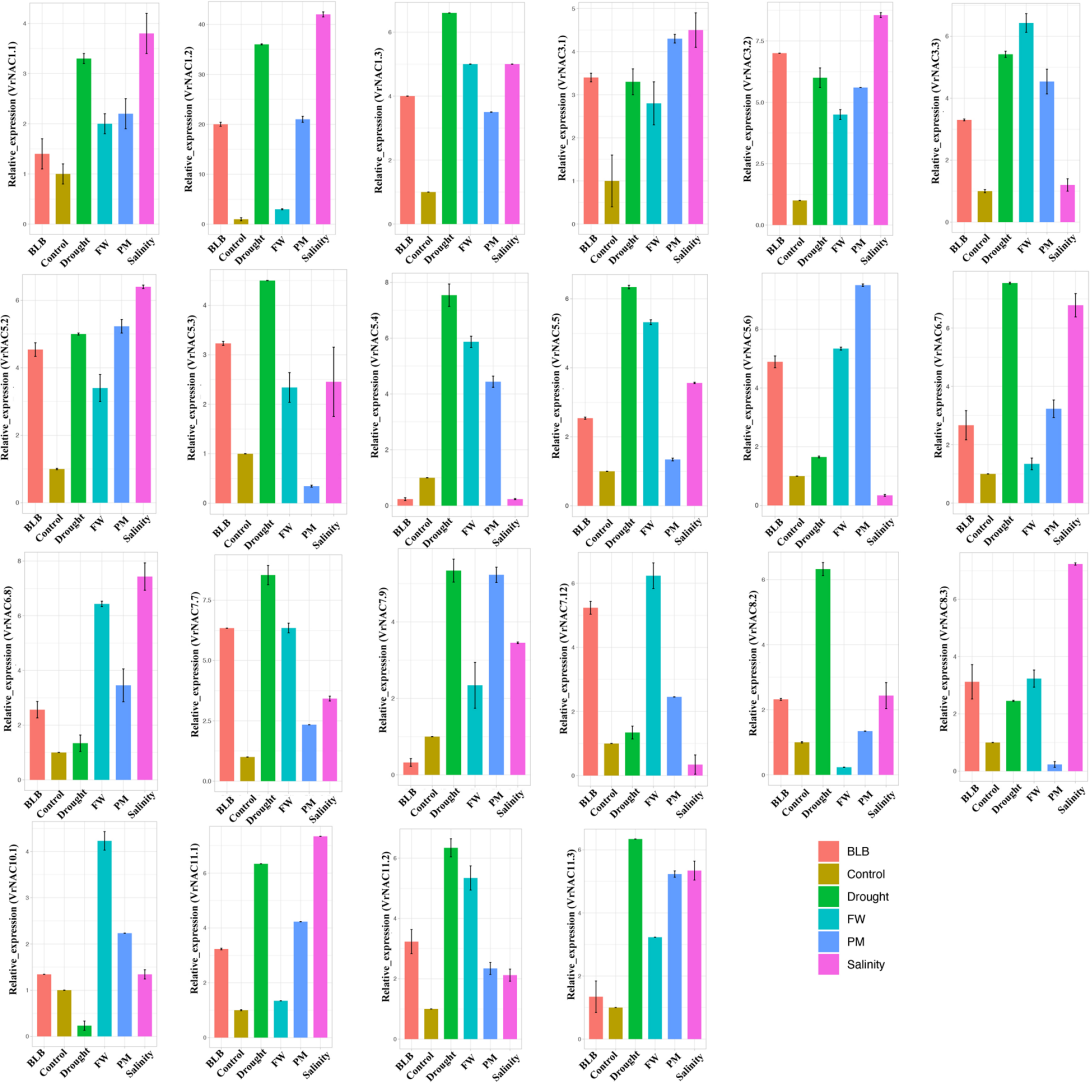
Bar plots exhibiting the qRT-PCR based gene expression pattern of 22 randomly selected *VrNAC* genes in response to abiotic or biotic stresses. Ubiquitin was used as an internal control in all reactions. Different colored bars represent expression patterns of *VrNACs* in response to various abiotic or biotic stresses. Bacterial leaf blight (BLB), Fusarium wilt (FW), and Pearl millet (PM), respectively. Bar plots were constructed using the R packages ggplot2 and RColorBrewer

The expression patterns of 22 different *VrNAC* genes were evaluated under both abiotic and biotic stresses (Fig. 10). Two weeks old mung bean leaves were inoculated with powdery mildew, bacterial leaf spot and fusarium wilt to assess the expression of the *VrNAC* genes in response to biotic stress. To assess the effects of abiotic stresses, leaves were exposed to drought and salinity stresses. The NM-98 mung bean variety used in these experiments is thought to be tolerant to drought stress and bacterial leaf spot. The results suggest that *VrNAC*s appeared to be induced in response to the abiotic stress compared to biotic stresses (Fig. 10). We found that expression of *VrNAC*7.7 is induced under bacterial leaf spot, powdery mildew and drought stress early following the application of stress. The genes *VrNAC*3.2, *VrNAC*5.2, *VrNAC*5.6 are also induced under biotic stresses. Based on the observed expression patterns of the *VrNAC* genes, we conclude that *VrNAC* genes playing significant roles under both abiotic and biotic stresses through many different pathways.

## Discussion

Mung bean, a legume crop, is an important food source and is consumed as a replacement for meat owing to its high protein content. In addition to providing a source of food for consumption, mung bean cultivation also has positive environmental effects, for instance by contributing to soil fertility through nitrogen fixation. Whole-genome sequences of many food legumes, such as pigeon pea [35], chickpea [36], and adzuki bean [37], have recently been released and such whole-genome sequencing data is a valuable resource for comparative and evolutionary studies. Transcription factors (TFs) are the major focus in biological research owing to the fact that TFs regulate the expression of downstream genes and play key roles in different pathways that regulate numerous biological processes in plants. Currently, a large number of TFs belong to different families, regulating drought, salinity, low temperature, hormonal, and pathogenic reactions in plants have been identified, and these transcription factors may thus be involved in different mechanisms allowing plants to handle different stresses.

As an example, NAC TFs in plants are involved in plant development and senescence as well as in response to both abiotic and biotic stresses. The mung bean genome contains 81 predicted *VrNAC* genes, most of which to date have not been characterized in detail. The goal of the current study was to identify the orthologs of all mung bean NAC genes via comparative evolutionary relationship among NAC TFs from both mung bean and Arabidopsis. NAC TFs are known to be widely distributed in different plant species and have potential roles in regulating plant development, growth and stress responses [38]. The NAC TF family is one of the largest TFs families identified to date [39] and there are, for example, 117 NAC genes in Arabidopsis [11], 151 in rice [12], 79 in grape [13], 180 in apple [40], 152 in maize [41], 71 in chickpea [42], 96 in cassava [43], 185 in Asian pears [44], and 80 in tartary buckwheat [17]. Assessing the gene structure of the 81 *V*r*NAC* genes, we found that they contain between one to five introns. The intron numbers of *VrNAC*s are different from those identified in other plants, such as in rice 0–16 [12], 0–8 in poplar [14], and 0–9 in cotton [45], respectively. Interestingly, identified stress-responsive *NACs* in chickpea, pigeon pea, and groundnut have two introns [46].

The diversity of gene structures and conserved motifs seen in mung bean NACs also indicate that these TFs are highly functionally diverse [47]. Gene duplication events are known to have an imperative role in the evolution and expansion of gene families and gene duplication of NAC TFs has been observed in many plant species [48]. We found 27 pairs of genes that exhibited evidence for duplication events among the 81 *VrNAC*s and this may have contributed to the expansion of the NAC family in mung bean. As different proteins having similar sequences are predicted to have diversified functions, we analyzed the functions of the *VrNAC* TFs based on their placement in a phylogenetic tree of NAC proteins.

In addition, we identified orthologous pairs of NACs TFs between mung bean and Arabidopsis based on protein sequence similarity. The comparative phylogenetic analysis of Arabidopsis and mung bean NAC proteins showed that NACs could be classified into 13 groups or clades, named NAC-I to NAC-XIII. The NAC-IX group constituted the largest clade with 36 NAC proteins. Group-IX contains 11 *VrNAC* which are largely involved in the formation of photoassimilate, a compound synthesized by assimilation under light-dependent reaction [49]. In this group, VrNAC1.6, an ortholog of AT3G17730, and VrNAC5.5, an ortholog of AT1G54330.1, are both involved in sugar transport through phloem sieve element cells in plants [50]. We also identified transcriptional activators, involved in the induction of abscisic acid (ABA) responsive genes, such as AT1G32510.1 and its homologous *VrNAC7.4* [51]. Moreover, AT4G17980.1 and AT5G46590.1 trigger the ABA-inducible genes in response to dehydration and osmotic stresses that lead to stomatal closure to inhibit further water loss under dehydration conditions [52]. They act synergistically with ABF2, which acts as a positive component of glucose signal transduction [53, 54]. However, AT2G27300.1 is found to be an ortholog of Vradi0425s00070.1, activated by proteolytic cleavage through intramembrane proteolysis (RIP), and induces GA-mediated salt-responsive suppression in seed germination and flowering via FLOWERING LOCUS T (FT) [55].

Among the 33 NACs in the NAC-X groups, 16 belong to *VrNAC*s and 17 are representing the AtNAC proteins. In this group, we identified three orthologous groups of mung bean and Arabidopsis NACs. AT3G12910.1 is an ortholog of VrNAC5.6, which acts as a negative regulator of leaf senescence in Arabidopsis [51]. The AT1G79580.1 is an ortholog of *VrNAC*5.4, regulates root cap development that determines the growth trajectory and expedites the root penetration in the soil [55]. Likewise, *VrNAC*8.3 is an ortholog to AT1G71930.1, and AT1G71930.1 participate in the formation of vascular system by regulating the immature xylem vessel-specific genes expression [56]. In addition, AT1G71930.1 contributes also to secondary cell wall biosynthesis and modification and programmed cell death [57]. In group-XI, VrNAC7.6 is an ortholog of AT1G61110.1 which is associated with anther development and pollen production, essential for normal seed development [58]. Besides, AT1G01720.1 is an ortholog of *VrNAC*6.6, belongs to a large family of putative transcriptional activators with the NAC domain and known as ATAF1. ATAF1 is representing the uncharacterized plant-specific gene family encoding NAC transcription factors and is regulated in response to various external stimuli in Arabidopsis. It is also involved in resistance to the non-host biotrophic pathogen *Blumeria graminis* f. sp. *hordei* in Arabidopsis [59]. In group-XII, the AT1G56010.1 ortholog of *VrNAC*5.7, encodes a transcription factor participating in shoot apical meristem and auxin-mediated lateral root development [60]. The group-XIII contains 25 NAC proteins in which *VrNAC*5.3 grouped with AT1G76420.1. However, AT1G76420.1 regulates the shoot apical meristem formation during embryogenesis and organ separation [60]. Group IV has 17 NACs, including eight *VrNAC*s and nine NACs from Arabidopsis. In this group, AT1G25580 is ortholog of *VrNAC*11.3, encoding *Suppressor of Gamma Response* 1 (*SOG*1), a putative transcription factor governing multiple responses to DNA damage [61]. The given results exhibited that putative mung bean *NAC* TFs orthologs with Arabidopsis NACs might have similar functions as previous studies approved the functional similarities among orthologs of different plant species [62].

Cis-acting regulatory elements (CAREs) are critical gene structures in eukaryote genomes. CAREs determine transcriptional initiation and are characterized by having conserved motifs between 5 to 20 nucleotides long that are found upstream of the transcriptional start codon. In this study, 133 drought stress-responsive CAREs were identified in the putative *VrNAC* genes. Important CAREs detected were light response elements that are the most abundant CARE identified for *VrNACs*. Additionally, 21 auxin-responsive elements and 20 stress and defense-responsive elements were also identified. Several other promoter elements were also identified that are known to play key roles in various plant development and stress responses such as seed regulation, endosperm expression, gibberellin acid, and salicylic acid. The existence of different cis-regulatory elements in the promoters receive special consideration as they provide insights into gene regulation and plant signaling under stress conditions.

A co-expression network ensures the coordination expression of genes that have important roles in different cellular processes during plant development and differentiation. Deciphering the predicted co-expression networking needs extensive genomic approaches to elucidate the functional and structural characteristics of the *VrNACs* to improve the knowledge of mung bean genome. This novel genome information would ultimately be useful for breeders to target the respective genes and develop resilient plant genotypes.

## Conclusions

Abiotic and biotic stress affects mung bean with varying severity at different growth stages, which can result in moderate to severe yield loss. We identified 81 NAC genes in mung bean and detailed analyses identified phylogenetic relationships among the genes, their chromosomal locations, gene structure, conserved motifs from their promotors and the expression profiles of the putative *VrNAC* genes. The comparative phylogenetic revealed clusters of *VrNAC*s and identified orthologs in Arabidopsis as well as several paralogs. Collinearity analyses identified 58 and 26 *VrNAC* genes having homologous pairing with tomato and rice, respectively and highlights that these plant species evolved from a common ancestor. The research findings presented here provides a milestone for further research aimed at accelerating functional genomics and molecular breeding programs in mung bean. Having detailed knowledge of stress-responsive *VrNAC*s in mung bean will be a highly valuable resource for future molecular breeding in food legumes.

## Methods

### Sequence data retrieval

The nucleotide as well as protein sequences of mung bean NAC (*VrNAC*) genes were searched for and retrieved from the plant transcription factor database (http://planttfdb.gao-lab.org/) (Table S7; Table S8). The sequence information of all *VrNAC* genes was cross-checked with National Center for Biotechnology Information (NCBI) database by Basic Local Alignment Search Tool (BLAST).

The HMM file of the NAM domain (PF02365) was retrieved from the Pfam database and was used to assess the NAC family proteins in mung bean based on an e-value less than 0.001 using HMMER 3.0 (http://hmmer.org/). All *Arabidopsis thaliana* protein sequences for comparative analysis were retrieved from the TAIR database (www.arabidopsis.org/) (Table S9). We determined the molecular weights and isoelectric points for all identified proteins of *VrNAC*s using the Expasy server (www.expasy.org/).

### Gene structure and chromosome location

To illustrate the gene structure of all putative *VrNAC*s, we used the Gene Structure Display Server 2.0 (http://gsds.cbi.pku.edu.cn/). This tool uses coding sequences as input to generate gene structures. The location of each *VrNAC* gene was determine by their start and end position on each mung bean chromosome, and their graphical representation was made by TBtool (https://github.com/CJ-Chen/TBtools/releases).

### Syntenic evolutionary analysis

Orthologous and paralogous NAC genes between Arabidopsis and mung bean were illustrated with different lines using Circos (http://circos.ca/) [63]. Whole-genome sequences and annotation files of Arabidopsis, tomato and rice were downloaded from Phytozome v13.0 (https://phytozome-next.jgi.doe.gov/). Syntenic evolutionary analyses between different plant species were performed using the genome and annotation files as inputs to the Multiple Collinearity Scan Tool kit (MCScanX, https://github.com/wyp1125/MCScanX). The Dual Synteny Plot in MCScanX was employed to identify the homologous pairs between mung bean and two other plant species [64]. Collinearity maps between mung bean and Arabidopsis, tomato or rice were constructed in this way.

### Comparative phylogenetic analysis

MEGA X (http://www.megasoftware.net/) was used to construct the phylogenetic tree of the *VrNAC*s genes, and the *VrNACs* genes were divided into different groups as per the phylogenetic tree nodes. All protein sequences of VrNACs were initially aligned by using ClustalW (http://www.ebi.ac.uk/clustalw/) with the default parameters. Moreover, to assess the support for the protein classification, a phylogenetic analysis of the NAC protein sequences was performed using 1000 bootstrapping values. For comparative studies, 81 and 73 NAC protein sequences obtained from mung bean and Arabidopsis, respectively, were used. Briefly, all 154 NAC proteins were aligned with the ClustalW program using a BLOSUM protein weight matrix. All the other parameters were used with their default settings. A comparative phylogenetic tree was inferred from the aligned sequences using the Neighbor-Joining (NJ) scheme combined with bootstrapping to assess node robustness using MEGA X (https://www.megasoftware.net/). The Poisson correction method was used for computing the evolutionary distances of different amino acids substitutions per site.

### Conserved motif analysis

The classification of domains in the putative *VrNAC*s was performed using the MEME suite 4.11.1 (http://meme-suite.org/tools/meme). Several conserved motifs were identified using optimum search parameters with maximum number of motifs = 20; minimum sites per motif = 2).

### Putative promoter cis-acting element analysis

The upstream 1 kb from the transcription start site was retrieved for all *VrNAC* genes using the NCBI database in order to perform promoter analysis via PlantCARE tool (http://bioinformatics.psb.ugent.be/webtools/plantcare/html/). All identified cis-elements were then highlighted at their respective positions in the 1 kb region of each *VrNAC* gene upstream region.

### Co-expression network construction

The co-expression data of *VrNAC*s were downloaded from the STRING database (https://string-db.org/cgi/). Initially, we ranked correlated genes with a Pearson correlation coefficient (PCC) higher than 0.4. Afterward, we arranged the *VrNACs* and co-expressed genes as per the PCCs threshold value. The Cytoscape (https://cytoscape.org/index.html) software was used to construct the co-expression network between *VrNAC*s and other co-expressed genes.

### miRNA prediction in VrNACs

All miRNA sequences from mung bean were retrieved from the Plant microRNA Encyclopedia (PmiREN, https://www.pmiren.com/). Mung bean genome sequence data representing the *VrNAC*s were submitted as candidate genes to predict potential miRNAs by searching against the data retrieved from PmiREN using the psRNATarget Server with the default parameters (http://plantgrn.noble.org/psRNATarget/).

### Plant materials and growth conditions

Two weeks old mung bean seedlings, belonging to the variety NM-98, were selected for stress treatments. NM-98 has earlier been identified to be tolerant to drought and bacterial leaf spot in Pakistan and is currently cultivated in many regions in Pakistan [65]. Plants were grown in the greenhouse with three biological replications in pots and growth conditions were maintained at temperatures of 28/23 °C for day and night, respectively. Light intensity was maintained at 600 μmolm^−2^s^−1^with day and night cycles of 14/10 h. For the salt treatment, 2 weeks old seedlings were transferred to Yoshida’s solution containing 100 mM NaCl. For the drought stress treatment, irrigation was halted after germination, and seedlings were collected for RNA extraction after 4 weeks. In addition to the abiotic stresses, we also inoculated the mung bean leaves with powdery mildew, Fusarium and bacterial leaf spot. Samples were collected in three biological replicates 3 hours after inoculations. Plants grown under normal conditions and without any stress treatments were considered as control samples for comparisons. All collected samples were stored at − 80 °C prior to the next step. All experiments performed in this study comply with relevant institutional, national, and international guidelines and legislation.

### qRT-PCR based expression profiling

Different genes from the *VrNAC* family were selected to assess their expression patterns under drought and salinity stress. Primers were designed based on the transcript sequences using R package OpenPrimeR. A list of designed primers for qRT-PCR is given in Table S10. Total leaf RNA from both control and treated samples were isolated by TRIzol reagent, and purified RNA was employed for cDNA synthesis using a cDNA synthesis kit (TransGen, Beijing, China) as per the manufacturer’s protocols. Ubiquitin, a housekeeping gene, was an internal control during qRT-PCR. The thermal cycler conditions were fixed as 95 °C for 30 s, keep on by 45 cycles of 95 °C for 30 sec, 60 °C for 40 s, and 72 °C for 15 s. The relative expression levels of the putative genes were calculated using 2^−ΔΔCT^ method [66].

## Supplementary Information

**Additional file 1.**

**Additional file 2.**

## Data Availability

All data generated or analysed during this study are were obtained from publicly available databases as described in this published article and its supplementary information files.
