# Supplementary material for: Genome-wide analyses of the mung bean NAC gene family reveals orthologs, co-expression networking and expression profiling under abiotic and biotic stresses
Source: BMC Plant Biol. 2022 Jul 15;22:343. doi: 10.1186/s12870-022-03716-4 (PMC9284730; doi:10.1186/s12870-022-03716-4)

**Supplementary Figures Labels**

**Fig. S1** Cis-regulatory element analysis of *VrNAC* TFs. Different cis regulatory boxes of different environmental stress response and different hormones are identified, mentioned in different colours.


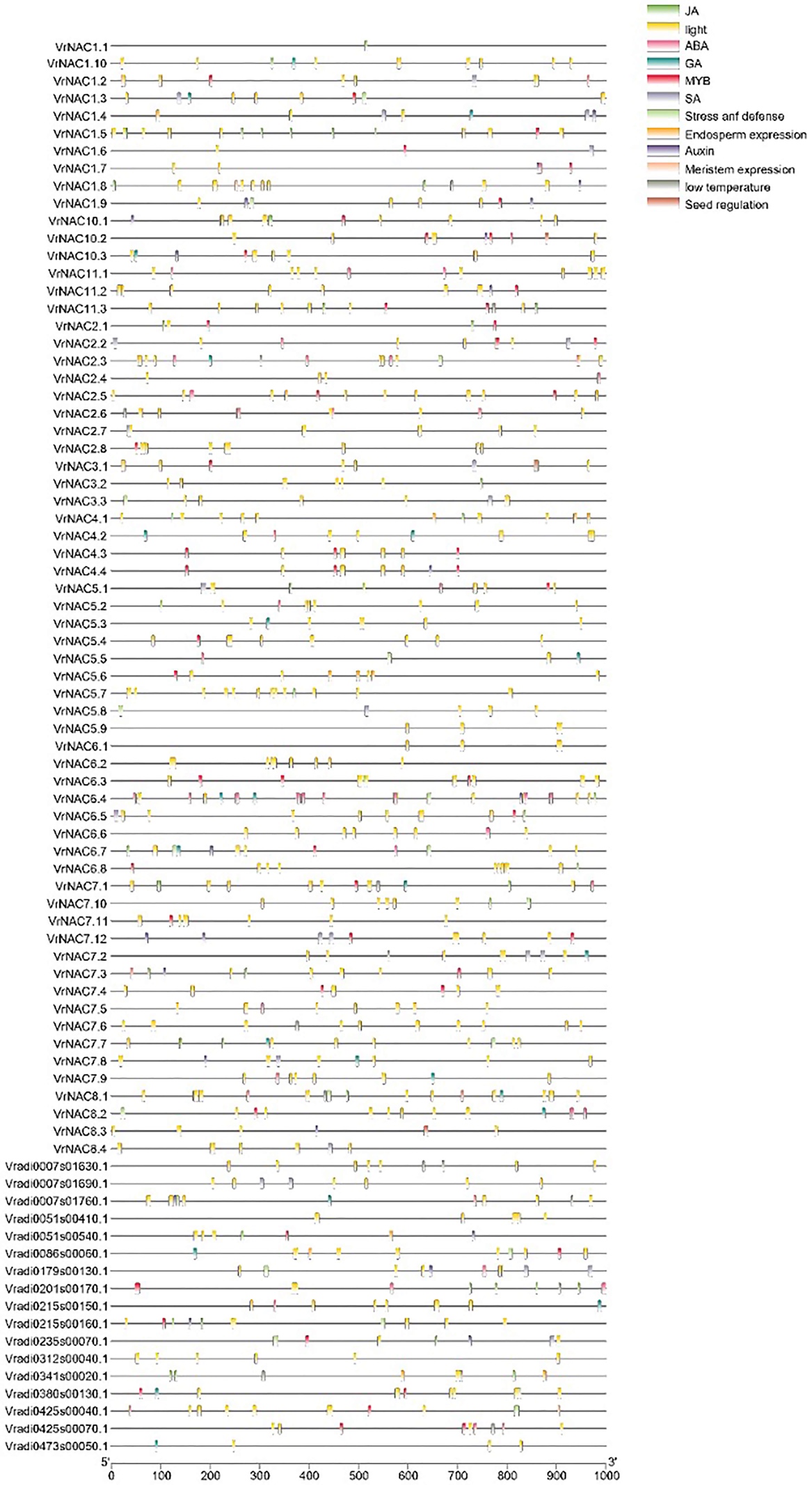

Supplement: Supplementary file 2 — Additional file 2. [file 12870_2022_3716_MOESM2_ESM.docx]
